# Correlation between coagulation abnormalities and *in vitro* fertilization outcomes in infertile patients with endometriosis: a retrospective cohort study

**DOI:** 10.3389/fmed.2026.1888342

**Published:** 2026-10-23

**Authors:** Xiufang Du, Wenjing Hu, Changna Fu

**Affiliations:** Shijiazhuang Maternal and Child Health Care Hospital, Shijiazhuang, Hebei, China

**Keywords:** antithrombin III, coagulation abnormality, d-dimer, endometriosis, *in vitro* fertilization, infertility

## Abstract

**Objective:**

To investigate coagulation function in infertile patients with endometriosis (EMs) undergoing IVF-ET and analyze its correlation with reproductive outcomes, providing evidence for perioperative optimization.

**Methods:**

This retrospective cohort study included 216 infertile patients (108 with EMs, 108 age-matched tubal-factor controls) undergoing IVF-ET from 2022 to 2024. Only the first IVF cycle and first embryo transfer per patient were included in the primary analysis to ensure independence of observations. Coagulation parameters (PT, APTT, TT, FIB, D-dimer, AT-III) were measured before controlled ovarian hyperstimulation. Coagulation abnormality was defined as the presence of at least one of the following: FIB > 4.0 g/L, D-dimer > 0.5 mg/L, or AT-III < 75%. PT, APTT, and TT were analyzed as continuous variables because no validated consensus threshold exists for defining shortened PT/APTT/TT as a hypercoagulable state. Baseline characteristics, COH parameters, IVF outcomes, and confounders were compared. EMs patients were stratified by this coagulation abnormality definition. Hierarchical logistic regression was used to identify factors associated with live birth failure.

**Results:**

EMs patients showed significantly shortened PT/APTT/TT, elevated FIB and D-dimer, and decreased AT-III when these parameters were analyzed as continuous variables (all *P* < 0.05); however, PT/APTT/TT were not used to classify patients as having coagulation abnormality. Based on the composite definition including FIB, D-dimer, and AT-III, the incidence of coagulation abnormalities was 48.15% (52/108) in the EMs group versus 7.41% (8/108) in the control group (*P* < 0.001). Among EMs patients, those with coagulation abnormalities had lower high-quality embryo rate, implantation rate, and live birth rate, and higher early abortion rate (all *P* < 0.05). Live birth rates decreased progressively across 0, 1, 2, and 3 abnormal parameters among FIB, D-dimer, and AT-III (44.6%, 29.6%, 16.7%, and 0.0%, respectively; P-trend = 0.002). In fully adjusted models, elevated D-dimer (OR = 2.864, 95% CI: 1.427–5.748, *P* = 0.003) and decreased AT-III (OR = 0.512, 95% CI: 0.276–0.949, *P* = 0.034) were independently associated with live birth failure, while high-quality embryo rate was protective (OR = 0.326, 95% CI: 0.154–0.689, *P* = 0.003). After additional adjustment for high-quality embryo rate, the association between elevated D-dimer and live birth failure was attenuated but remained statistically significant. This attenuation was interpreted as a change in the adjusted association rather than as evidence of causality. Fibrinogen was not significant (*P* = 0.227). In multivariable models, elevated D-dimer and decreased AT-III were independently associated with live birth failure after adjustment for embryo quality. Subgroup analysis suggested stronger associations in stage III–IV EMs, but this finding was exploratory.

**Conclusion:**

Coagulation abnormalities, defined by FIB, D-dimer, and AT-III, were more frequent in infertile EMs patients and were associated with adverse IVF outcomes. Elevated D-dimer and decreased AT-III were independently associated with live birth failure. These findings are exploratory; routine D-dimer and AT-III testing cannot currently be recommended until prospective validation is available.

## 1 Introduction

Endometriosis (EMs) is a common benign gynecological estrogen-dependent chronic disease characterized by the growth of endometrial glands and stroma outside the uterine cavity, with an incidence of 6%–10% in women of childbearing age (1, 2). Notably, the prevalence of EMs among infertile patients is as high as 30%–50%, and 40%–50% of patients with EMs experience infertility, making it one of the leading causes of female infertility and significantly impacting patients' physical and mental health as well as reproductive quality (3, 4). For EMs-related infertility, *in vitro* fertilization-embryo transfer (IVF-ET) represents the first-line assisted reproductive technology (1, 5, 6). However, clinical observations indicate that compared with other causes of infertility (e.g., tubal factor), patients with EMs exhibit lower implantation rates, higher early pregnancy loss, and reduced live birth rates following IVF treatment, with the underlying mechanisms not yet fully elucidated (3, 7, 8).

In recent years, the relationship between EMs and coagulation dysfunction has garnered increasing attention. Accumulating evidence suggests that EMs is not merely a localized pelvic condition but also a systemic chronic inflammatory disease (9–11). Persistent low-grade systemic inflammation may activate the coagulation cascade, inhibit fibrinolysis, and induce a prothrombotic state (12, 13). Coagulation dysfunction may impair ovarian and endometrial microcirculation, potentially compromising follicular development and endometrial receptivity (14–16). Furthermore, it may increase the risk of early miscarriage after embryo implantation.

To date, most studies have focused on the association between EMs and hypercoagulability, but systematic investigations examining the relationship between coagulation abnormalities and IVF outcomes in EMs-related infertility remain limited, with insufficient clinical evidence (12, 17). Therefore, this retrospective cohort study aimed to systematically evaluate coagulation profiles in infertile patients with EMs undergoing IVF and to examine whether the association between coagulation abnormalities and key IVF outcomes remains after adjustment for embryo quality, with the goal of providing preliminary evidence for future research on perioperative risk stratification and management in this population.

## 2 Materials and methods

### 2.1 Study design and participants

This retrospective cohort study was conducted at the Reproductive Medicine Center of Shijiazhuang Maternal and Child Health Care Hospital between January 2022 and December 2024. The study was approved by the Medical Ethics Committee of Shijiazhuang Maternal and Child Health Care Hospital (Approval No. 202224) and was performed in accordance with the Declaration of Helsinki (2013 revision). The requirement for written informed consent was waived by the Ethics Committee because of the retrospective and anonymized nature of the study.

Inclusion Criteria: (1) Age 22–38 years, duration of infertility ≥1 year; (2) Diagnosis of EMs confirmed by laparoscopy. Preoperative clinical symptoms, transvaginal ultrasound, pelvic MRI, and serum CA125 levels were used as supportive information but were not used as the sole basis for diagnosis or staging. Staging was assigned according to the revised American Fertility Society (r-AFS) classification based on surgical records. (3) Normal ovarian reserve: basal follicle-stimulating hormone (bFSH) < 10 IU/L, anti-Mullerian hormone (AMH) ≥ 1.2 ng/mL, antral follicle count (AFC) ≥ 5; (4) Complete clinical and follow-up data.(5) For the control group: tubal-factor infertility only, with no other infertility factors; no history of EMs, adenomyosis, or autoimmune diseases. EMs was excluded by medical history, absence of typical symptoms, transvaginal ultrasound, and pelvic MRI. Laparoscopy was not routinely performed in the control group; therefore, subclinical or asymptomatic EMs cannot be completely excluded.

Exclusion Criteria: (1) Coexisting adenomyosis, uterine fibroids, endometrial polyps, or other organic uterine diseases; (2) Autoimmune diseases (e.g., systemic lupus erythematosus, antiphospholipid syndrome), hematological disorders, liver or renal dysfunction, or malignancies; (3) Use of anticoagulant or antiplatelet medications within 3 months before treatment; (4) History of recurrent spontaneous abortion (≥2) or prior IVF failure; (5) Incomplete clinical or follow-up data.

### 2.2 Diagnosis and staging of endometriosis

All 108 patients with EMs were diagnosed by laparoscopy. No patient was diagnosed by imaging alone. The diagnosis was based on laparoscopic findings, and pathological confirmation was available where clinically indicated. Stage was assigned according to the revised American Fertility Society (r-AFS) classification based on surgical records: 42 patients (38.89%) had stage I–II disease and 66 patients (61.11%) had stage III–IV disease. Preoperative transvaginal ultrasound, pelvic MRI, and serum CA125 were used as supportive clinical information but were not used to assign r-AFS stage.

### 2.3 Control group

The control group comprised 108 age-matched infertile women with tubal-factor infertility only. EMs was excluded by medical history, absence of typical symptoms, transvaginal ultrasound, and pelvic MRI. Laparoscopy was not routinely performed in the control group; therefore, subclinical or asymptomatic EMs cannot be completely excluded. Coagulation status was not an inclusion or exclusion criterion for either group. Coagulation parameters were measured after enrollment and before COH, and were classified *post hoc* according to the prespecified reference ranges. Therefore, the presence of coagulation abnormalities among controls reflects the background prevalence in this population and did not influence control selection.

### 2.4 Unit of analysis

Only the first IVF cycle and the first embryo transfer per patient were included in the primary analysis to ensure independence of observations. Patients who underwent more than one cycle or transfer were included only once. Each patient therefore contributed a single IVF cycle and a single embryo transfer, and observations were independent. No repeated-measures adjustment was required.

### 2.5 Research methods

#### 2.5.1 Coagulation function assessment

##### 2.5.1.1 Blood collection and measurement

Fasting peripheral venous blood (5 mL) was collected from all patients on days 2–3 of the menstrual cycle, before initiation of controlled ovarian hyperstimulation (COH). Samples were centrifuged at 3000 rpm for 10 min, and the supernatant was analyzed using an automated coagulation analyzer (Sysmex CS-5100, Sysmex Corporation, Kobe, Japan). The following parameters were measured: prothrombin time (PT), activated partial thromboplastin time (APTT), thrombin time (TT), fibrinogen (FIB), D-dimer (D-D), and antithrombin III (AT-III) activity. Reagents were obtained from Siemens Healthcare Diagnostics (Marburg, Germany): Dade Innovin for PT, Dade Actin FSL for APTT, Thromboclotin for TT, Dade Thrombin Reagent for FIB, INNOVANCE D-Dimer for D-D, and INNOVANCE Antithrombin for AT-III. All assays were performed according to the manufacturers' instructions, and the laboratory participates in regular external quality assessment.

Reference ranges: The laboratory reference ranges used in this study were: PT 11.0–13.0 s, APTT 25.0–35.0 s, TT 14.0–21.0 s, FIB 2.0–4.0 g/L, D-D 0–0.5 mg/L, and AT-III 75%–125%. These ranges were established by the clinical laboratory of Shijiazhuang Maternal and Child Health Care Hospital and are consistent with the reagent manufacturers' recommended ranges for the Sysmex CS-5100 platform.

Definition of coagulation abnormalities: Primary definition. Coagulation abnormality was defined as the presence of at least one of the following: FIB > 4.0 g/L, D-dimer > 0.5 mg/L, or AT-III < 75%. PT, APTT, and TT were measured and analyzed as continuous variables and are reported descriptively, but were not included in the composite definition of coagulation abnormality because no internationally standardized cutoff exists for defining shortened PT/APTT/TT as a hypercoagulable state.

PT, APTT, and TT as continuous variables. Shortened PT, APTT, and TT have been consistently reported in women with endometriosis. A recent systematic review and meta-analysis of 22 studies including 6,258 participants confirmed that APTT (SMD = −0.13, *P* = 0.010), PT (SMD = −0.31, *P* = 0.009), and TT (SMD = −0.41, *P* < 0.001) are significantly shortened in endometriosis compared with controls (12). Ling and Wang similarly found that APTT, PT, and TT were shortened in endometriosis and were prolonged after treatment (18). These findings support the biological rationale for examining PT/APTT/TT as continuous markers of a hypercoagulable phenotype in endometriosis. However, because no validated consensus threshold exists for defining shortened PT/APTT/TT, these parameters were not included in the primary composite definition of coagulation abnormality.

Rationale for using “at least one abnormality” as the primary definition. Because coagulation activation in endometriosis is likely a continuum rather than a dichotomous state, and because different patients may exhibit abnormalities in different components of the hemostatic system, a composite definition based on at least one of three abnormal parameters (FIB, D-dimer, AT-III) was used to maximize sensitivity for detecting a prothrombotic tendency. This approach is consistent with the exploratory aim of the study, which was to identify patients who might warrant further coagulation evaluation before IVF.

Sensitivity analyses. Because the primary definition may group together patients with mild isolated derangements and those with more severe combined abnormalities, the following prespecified sensitivity analyses were performed: (1) a stricter definition requiring at least two concurrent abnormalities among the three parameters (FIB, D-dimer, AT-III); (2) a composite coagulation score based on the number of abnormal parameters (0, 1, 2, or 3), analyzed as an ordinal variable to explore dose-response relationships; and (3) the individual coagulation markers analyzed as continuous variables. These analyses were intended to assess whether the association with IVF outcomes was robust to the definition of coagulation abnormality.

#### 2.5.2 IVF treatment protocol and data collection

All patients underwent COH using either the conventional long protocol or the antagonist protocol based on age and ovarian reserve. When dominant follicles reached 18–20 mm, human chorionic gonadotropin (HCG) was administered to trigger ovulation, and oocyte retrieval was performed under transvaginal ultrasound guidance 36–38 h later. Fertilization was achieved via conventional IVF or intracytoplasmic sperm injection (ICSI) according to semen parameters. Embryos were cultured to the cleavage stage (day 3) or blastocyst stage (days 5–6), and either fresh or frozen-thawed embryo transfer was performed based on endometrial status and OHSS risk. Luteal phase support was routinely provided after transfer.

The following variables were recorded: baseline characteristics, coagulation parameters, COH parameters, key confounding variables (COH protocol, fertilization method, embryo transfer stage, transfer type, endometrial thickness, number of embryos transferred), and IVF outcomes (fertilization rate, cleavage rate, high-quality embryo rate, implantation rate, clinical pregnancy rate, early abortion rate, live birth rate). Each patient contributed one IVF cycle and one embryo transfer to the primary analysis; no patient contributed more than one eligible cycle.

### 2.6 Statistical analysis

Statistical analyses were performed using SPSS 26.0 and R software (version 4.2.0). Normally distributed continuous variables were expressed as mean ± standard deviation and compared using independent samples t-tests. Non-normally distributed variables were expressed as median (interquartile range) and compared using the Mann–Whitney U test. Categorical variables were expressed as frequencies (%) and compared using the chi-square test or Fisher's exact test as appropriate.

The primary analysis used multivariable logistic regression with live birth failure as the dependent variable. Only the first IVF cycle and first embryo transfer per patient were included to ensure independence of observations. Three hierarchical models were constructed. Model 1 included age, BMI, EMs stage, COH protocol, embryo transfer stage, and transfer type. Model 2 additionally included D-dimer and AT-III. Model 3 additionally included high-quality embryo rate. Variables with *P* < 0.10 in univariate analysis were eligible for entry. Because the study was not designed to establish causal pathways, changes in the adjusted odds ratios after adding high-quality embryo rate were examined descriptively and were not interpreted as evidence of causality. The variance inflation factor (VIF) was used to assess multicollinearity, and VIF < 2 was considered acceptable.

Subgroup analysis was stratified by EMs stage (I–II vs. III–IV), with a formal stage × coagulation status interaction test. Sensitivity analyses included: (1) excluding extreme coagulation values (D-dimer > 3.0 or < 0.2 mg/L); (2) stricter definitions of coagulation abnormalities (≥2 concurrent abnormalities among FIB, D-dimer, and AT-III) and a composite coagulation score (0, 1, 2, 3) as an ordinal variable; (3) coagulation markers as continuous variables; and (4) excluding controls with elevated CA125 (> 35 U/mL) or TVUS/MRI findings suggestive of EMs. D-dimer and fibrinogen were compared using adjusted ORs from separate fully adjusted models. A two-tailed *P* < 0.05 was considered statistically significant.

## 3 Results

### 3.1 Participant flow and baseline characteristics

During the study period, 241 patients were screened for eligibility, and 19 were excluded before enrollment (reasons are detailed in Figure 1). The remaining 222 patients initiated controlled ovarian hyperstimulation (COH). Among them, 3 developed moderate-to-severe OHSS and 3 had cycle cancellation for other reasons; these patients were excluded according to the prespecified exclusion criteria because they did not undergo embryo transfer. The final analytic cohort therefore included 216 patients (108 with EMs and 108 age-matched tubal-factor controls) who completed COH and underwent embryo transfer. Each patient contributed one IVF cycle and one embryo transfer to the primary analysis. The participant flow is shown in Figure 1.

**Figure 1:**
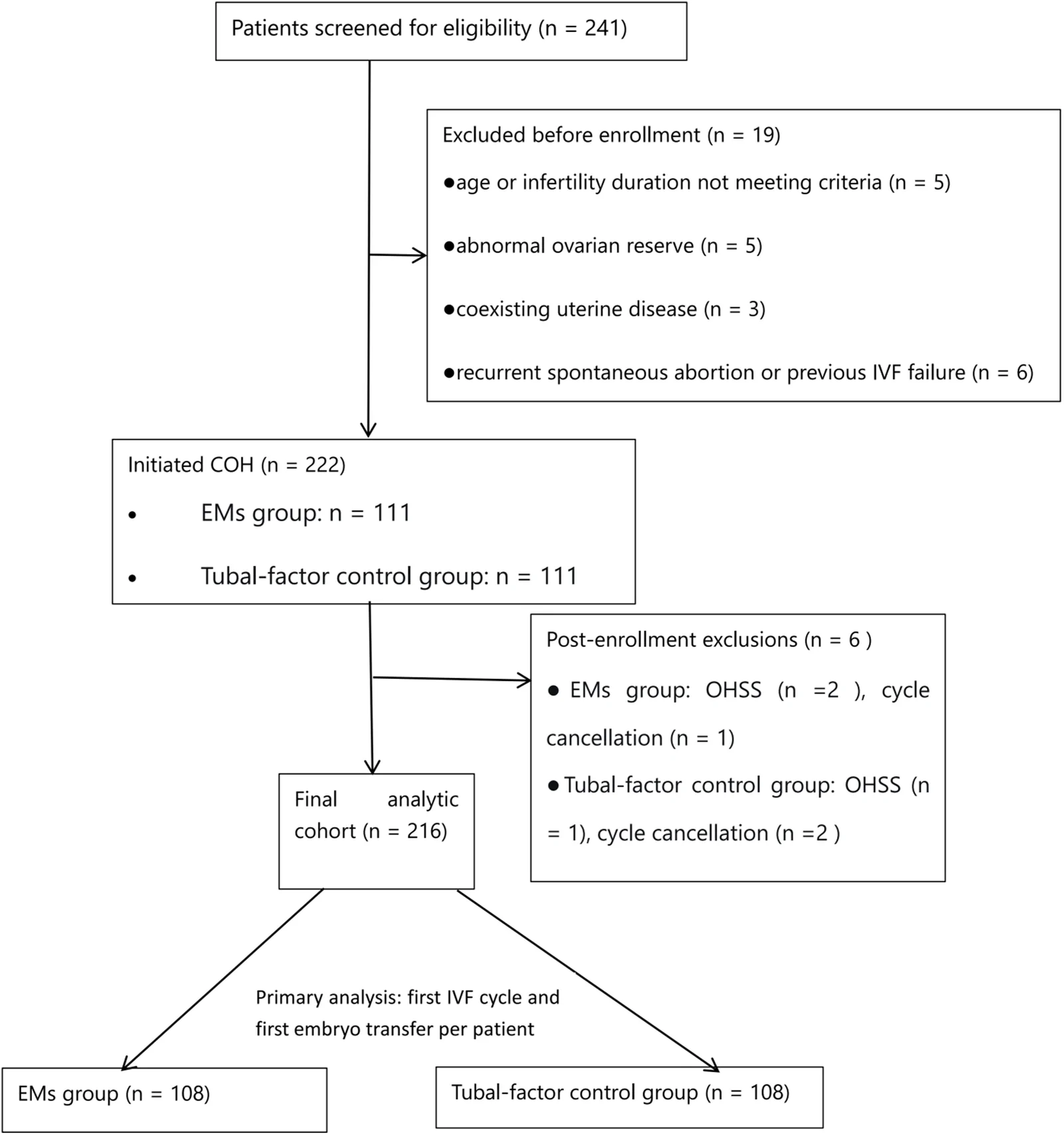
Participant flow diagram. Patients screened for eligibility (*n* = 241); excluded before enrollment (*n* = 19); initiated COH (*n* = 222); post-enrollment exclusions (*n* = 6: OHSS *n* = 3, cycle cancellation *n* = 3); final analytic cohort (*n* = 216; EMs *n* = 108, tubal-factor control *n* = 108). Only the first IVF cycle and first embryo transfer per patient were included in the analysis. COH, controlled ovarian hyperstimulation; EMs, endometriosis; OHSS, ovarian hyperstimulation syndrome.

No significant differences were observed between the EMs and control groups in age, BMI, infertility duration, bFSH, AMH, or AFC (*P* > 0.05), indicating balanced baseline characteristics. Serum CA125 levels were significantly higher in the EMs group (*P* < 0.001). All 108 EMs patients were diagnosed by laparoscopy; no patient was diagnosed by imaging alone. According to surgical r-AFS staging, 42 patients (38.89%) had stage I–II disease and 66 patients (61.11%) had stage III–IV disease. EMs stage was not applicable to the control group because these women had no endometriosis. Key confounding variables, including COH protocol, fertilization method, embryo transfer stage, transfer type, endometrial thickness, and number of embryos transferred, were comparable between groups (all *P* > 0.05), suggesting that treatment-related factors were balanced between groups (Table 1).

**Table 1:** Baseline and clinical characteristics of the study and control groups.

Characteristic	Study Group (EMs, *n* = 108)	Control Group (Tubal Infertility, *n* = 108)	t/*χ*^2^	*P*-value
Age (years)	31.24 ± 3.56	30.98 ± 3.42	0.542	0.588
BMI (kg/m^2^)	22.65 ± 2.31	22.48 ± 2.27	0.561	0.575
Infertility duration (years)	4.12 ± 1.65	3.98 ± 1.59	0.647	0.518
bFSH (IU/L)	6.85 ± 1.42	6.79 ± 1.38	0.326	0.745
AMH (ng/mL)	3.26 ± 1.15	3.31 ± 1.18	0.315	0.753
AFC (n)	12.65 ± 3.42	12.71 ± 3.38	0.136	0.892
CA125 (U/mL)	48.62 ± 12.53	16.35 ± 5.26	24.651	<0.001
EMs stage (I–II/III–IV), *n* (%)				
Stage I–II	42 (38.89)	–	–	–
Stage III–IV	66 (61.11)	–	–	–
Diagnostic method, *n* (%)				
Laparoscopy	108 (100.0)			
COH protocol (long/antagonist), *n* (%)	62 (57.41)/46 (42.59)	64 (59.26)/44 (40.74)	0.076	0.783
Fertilization method (IVF/ICSI), *n* (%)	72 (66.67)/36 (33.33)	75 (69.44)/33 (30.56)	0.214	0.644
Transfer stage (cleavage/blastocyst), *n* (%)	58 (53.70)/50 (46.30)	60 (55.56)/48 (44.44)	0.079	0.779
Transfer type (fresh/frozen), *n* (%)	49 (45.37)/59 (54.63)	51 (47.22)/57 (52.78)	0.075	0.784
Endometrial thickness (mm)	9.86 ± 1.25	9.92 ± 1.21	0.354	0.724
Transferred embryos (n)	2.01 ± 0.12	2.03 ± 0.11	1.265	0.207

All 108 EMs patients were diagnosed by laparoscopy; no patient was diagnosed by imaging alone. r-AFS stage was assigned according to the revised American Fertility Society (r-AFS) classification based on surgical records. EMs stage was assessed only in the EMs group because the control group had no endometriosis; therefore, staging was not applicable. All EMs patients were diagnosed by laparoscopy, and r-AFS stage was assigned based on surgical records.

### 3.2 Coagulation profiles

Patients with EMs demonstrated significantly shortened PT, APTT, and TT, elevated FIB and D-dimer levels, and reduced AT-III activity compared with controls (all *P* < 0.05). When PT, APTT, and TT were analyzed as continuous variables, these differences were consistent with a hypercoagulable phenotype; however, they were not used to classify patients as having coagulation abnormality. Based on the composite definition including FIB, D-dimer, and AT-III, the incidence of coagulation abnormalities was 48.15% (52/108) in the EMs group versus 7.41% (8/108) in the control group (*χ*^2^ = 42.704, *P* < 0.001) (Table 2).

**Table 2:** Comparison of coagulation parameters between groups.

Parameter	EMs Group (*n* = 108)	Control Group (*n* = 108)	t/Z	*P*-value
PT (s)	10.26 ± 0.85	11.85 ± 0.92	13.264	<0.001
APTT (s)	22.35 ± 2.16	28.69 ± 2.43	19.532	<0.001
TT (s)	12.68 ± 1.32	17.56 ± 1.58	23.651	<0.001
FIB (g/L)	4.52 ± 0.76	3.15 ± 0.62	14.237	<0.001
D-D (mg/L)	1.92 ± 0.68	0.36 ± 0.15	22.695	<0.001
AT-III (%)	68.52 ± 6.35	96.35 ± 7.26	29.413	<0.001
Coagulation abnormalities, *n* (%)	52 (48.15%)	8 (7.41%)	44.68	<0.001

Coagulation abnormality was defined as the presence of at least one of the following: FIB > 4.0 g/L, D-dimer > 0.5 mg/L, or AT-III < 75%. PT, APTT, and TT were analyzed as continuous variables and were not included in the composite definition of coagulation abnormality because no internationally standardized cutoff exists threshold exists for defining shortened PT/APTT/TT as a hypercoagulable state.

### 3.3 Subgroup analysis by coagulation status in EMs patients

Among EMs patients, 52 were classified into the coagulation abnormality subgroup and 56 into the normal coagulation subgroup according to the composite definition based on FIB, D-dimer, and AT-III. No significant differences were found between subgroups regarding age, ovarian reserve parameters, or COH characteristics (all *P* > 0.05). Regarding IVF outcomes, the coagulation abnormality subgroup showed significantly lower high-quality embryo rate, implantation rate, and live birth rate, and a significantly higher early abortion rate compared with the normal coagulation subgroup (all *P* < 0.05). Fertilization and cleavage rates did not differ significantly between subgroups (Table 3).

**Table 3:** Subgroup analysis of IVF outcomes by coagulation status in ems patients.

Outcome	Coagulation abnormality (*n* = 52)	Normal coagulation (*n* = 56)	t/χ^2^	*P*-value
Fertilization rate (%)	73.8 ± 11.1	76.5 ± 10.8	1.274	0.205
Cleavage rate (%)	91.7 ± 6.6	93.4 ± 6.1	1.341	0.182
High-quality embryo rate (%)	43.1 ± 13.8	54.7 ± 14.2	4.332	<0.001
Implantation rate (%)	26.2	39.7	5.791	0.016
Clinical pregnancy rate, *n* (%)	19 (36.5%)	31 (55.4%)	3.84	0.050
Early abortion rate, *n* (%)	8 (42.1%)	5 (16.1%)	4.13	0.042
Live birth rate, *n* (%)	11 (21.2%)	25 (44.6%)	6.69	0.010

Coagulation abnormality was defined as the presence of at least one of the following: FIB > 4.0 g/L, D-dimer > 0.5 mg/L, or AT-III < 75%. PT, APTT, and TT were analyzed as continuous variables and were not included in the composite definition of coagulation abnormality because no validated consensus threshold exists for defining shortened PT/APTT/TT as a hypercoagulable state.

### 3.4 Multivariate logistic regression

Hierarchical logistic regression was performed to examine the independent contribution of coagulation markers to live birth failure and whether these associations persisted after accounting for embryo quality. The results of the hierarchical logistic regression models are presented in Table 4. In Model 1 (clinical baseline: age, BMI, EMs stage, COH protocol, embryo transfer stage, and transfer type), advanced-stage EMs (stage III–IV) was associated with live birth failure (OR = 1.874, 95% CI: 1.006–3.492, *P* = 0.048), whereas age (OR = 1.058, 95% CI: 0.975–1.148, *P* = 0.172), BMI (OR = 1.045, 95% CI: 0.958–1.140, *P* = 0.316), antagonist protocol (OR = 1.298, 95% CI: 0.682–2.471, *P* = 0.426), blastocyst transfer (OR = 0.638, 95% CI: 0.341–1.194, *P* = 0.158), and frozen transfer (OR = 1.162, 95% CI: 0.617–2.189, *P* = 0.644) were not independently associated with the outcome.

**Table 4:** Hierarchical logistic regression models for live birth failure in ems patients.

Variable	Model 2: OR (95% CI)	*P*	Model 3: OR (95% CI)	*P*
Age (years)	1.061 (0.977-1.153)	0.157	1.044 (0.958-1.137)	0.327
BMI (kg/m^2^)	1.049 (0.961-1.146)	0.278	1.031 (0.942-1.128)	0.521
EMs stage III-IV (vs. I-II)	1.942 (1.024-3.684)	0.042	1.763 (0.914-3.401)	0.091
COH protocol: antagonist (vs. long)	1.347 (0.698-2.600)	0.374	1.210 (0.614-2.385)	0.582
D-dimer (elevated or per 1 mg/L)	3.412 (1.683-6.917)	0.001	2.864 (1.427-5.748)	0.003
AT-III (decreased or per 1%)	0.452 (0.237-0.862)	0.015	0.512 (0.276-0.949)	0.034
High-quality embryo rate	Not included	—	0.326 (0.154-0.689)	0.003
Embryo transfer stage: blastocyst	0.614 (0.323-1.167)	0.137	0.672 (0.347-1.301)	0.237
Transfer type: frozen	1.217 (0.632-2.341)	0.556	1.124 (0.573-2.204)	0.733

Model 1, clinical baseline model; Model 2, clinical baseline plus D-dimer and AT-III; Model 3, post-embryology model additionally including high-quality embryo rate.

OR, odds ratio; CI, confidence interval; EMs, endometriosis; COH, controlled ovarian hyperstimulation; AT-III, antithrombin III.

In Model 2, which additionally included D-dimer and AT-III, elevated D-dimer (OR = 3.412, 95% CI: 1.683–6.917, *P* = 0.001) and decreased AT-III (OR = 0.452, 95% CI: 0.237–0.862, *P* = 0.015) were independently associated with live birth failure. In Model 3, which additionally included high-quality embryo rate, elevated D-dimer (OR = 2.864, 95% CI: 1.427–5.748, *P* = 0.003) and decreased AT-III (OR = 0.512, 95% CI: 0.276–0.949, *P* = 0.034) remained independently associated with live birth failure, and high-quality embryo rate was protective (OR = 0.326, 95% CI: 0.154–0.689, *P* = 0.003). The OR for D-dimer was attenuated by 16.1% from Model 2 to Model 3 but remained statistically significant, indicating that the association was not fully explained by embryo quality. This attenuation was interpreted as a change in the adjusted association rather than as evidence of causality.

### 3.5 Subgroup and sensitivity analyses

Subgroup analysis stratified by EMs stage suggested that the association between coagulation abnormality and live birth failure was more pronounced in patients with stage III–IV EMs (OR = 3.214, 95% CI: 1.523–6.782, *P* = 0.002) than in those with stage I–II disease (OR = 1.876, 95% CI: 0.876–4.015, *P* = 0.108). However, the formal stage × coagulation status interaction test was *P* = 0.142; not statistically significant, and this subgroup finding should therefore be considered exploratory.

Sensitivity analyses showed that when D-dimer and AT-III were analyzed as continuous variables, both remained associated with live birth failure (D-dimer: OR = 1.721, 95% CI: 1.234–2.399, *P* = 0.001; AT-III: OR = 0.963, 95% CI: 0.933–0.994, *P* = 0.019). Exclusion of patients with extreme coagulation values did not materially alter the results (D-dimer: OR = 2.473, 95% CI: 1.187–5.153; AT-III: OR = 0.541, 95% CI: 0.289–0.999). After excluding controls with elevated CA125 or TVUS/MRI findings suggestive of EMs, the results were essentially unchanged. These sensitivity analyses are summarized in Table 5.

**Table 5:** Sensitivity analyses for the association between coagulation parameters and live birth failure**.**

Sensitivity analysis scenario	Variable	Adjusted OR (95% CI)	*P*-value
1. Coagulation markers as continuous variables	D-dimer (per 1 mg/L increment)	1.721 (1.234-2.399)	0.001
	AT-III (per 1% increment)	0.963 (0.933-0.994)	0.019
2. Excluding extreme coagulation values(D-dimer > 3.0 or <0.2 mg/L, *n* = 96)	Elevated D-dimer	2.473 (1.187-5.153)	0.016
	Decreased AT-III	0.541 (0.289-0.999)	0.049
3. Strict coagulation abnormality definition (≥2 abnormal parameters, *n* = 25)	≥2 abnormal coagulation markers	3.116 (1.344–7.216)	0.008
4. Composite coagulation score (ordinal: 0/1/2/3)	Per one-point increase in abnormal-parameter count	1.641 (1.184–2.274)	0.003
5. Excluding potential EMs suspects in control group(restricted EMs-case analysis only)	Elevated D-dimer	2.741 (1.351-5.563)	0.005
	Decreased AT-III	0.533 (0.284-0.998)	0.049

Sensitivity analyses included continuous coagulation variables, exclusion of extreme coagulation values (D-dimer >3.0 or <0.2 mg/L), a strict definition of coagulation abnormalities (≥2 concurrent abnormalities among FIB, D-dimer, and AT-III), a composite coagulation score (0, 1, 2, 3), and exclusion of controls with elevated CA125 (>35 U/mL) or TVUS/MRI findings suggestive of EMs.

OR, odds ratio; CI, confidence interval; EMs, endometriosis; AT-III, antithrombin III.

When a stricter definition of coagulation abnormalities (≥2 concurrent abnormalities among FIB, D-dimer, and AT-III) was applied, 25 (23.15%) of 108 EMs patients were classified as having coagulation abnormalities. The live birth rate was lower in this group than in the remaining patients (12.0% vs. 39.7%, *P* = 0.007), with an adjusted association with live birth failure (OR = 3.116, 95% CI: 1.344–7.216, *P* = 0.008). Live birth rate decreased across 0, 1, 2, and 3 abnormal coagulation parameters among FIB, D-dimer, and AT-III (0: 44.6%, 1: 29.6%, 2: 16.7%, 3: 0.0%; *P* for trend = 0.002). This gradient is consistent with a possible dose–response pattern, but it should be regarded as exploratory and requires confirmation.

## 4 Discussion

This study systematically assessed coagulation profiles among infertile patients with endometriosis undergoing IVF-ET and investigated their relationships with reproductive outcomes. Our principal findings were as follows: (1) a high prevalence of coagulation abnormalities defined by FIB, D-dimer, and AT-III was observed in patients with EMs (48.15%, 52/108); (2) such coagulation abnormalities were correlated with poorer embryo quality, reduced implantation and live-birth rates, and elevated risk of early pregnancy loss; and (3) elevated D-dimer and reduced AT-III represented independent risk factors for live-birth failure.

The prothrombotic state observed in EMs patients is consistent with the emerging understanding of EMs as a systemic inflammatory disorder. Chronic inflammation driven by ectopic endometrial lesions leads to sustained release of pro-inflammatory cytokines, which may activate the coagulation cascade, enhance fibrinogen synthesis, and suppress natural anticoagulants including AT-III (19, 20).

Our subgroup analysis revealed that coagulation abnormalities exerted no significant influence on fertilization or cleavage rates but were more strongly linked to downstream reproductive outcomes, including high-quality embryo formation, implantation, and live birth. These observations imply that the harmful impacts of coagulation abnormalities may predominantly impair endometrial receptivity and early placentation (21), instead of interfering with gamete interaction or early embryonic development. The apparently stronger association among patients with advanced-stage EMs requires cautious interpretation, given a non-significant formal interaction test (*P* = 0.142). This subgroup finding is hypothesis-generating and cannot confirm a dose-dependent biological mechanism (22, 23). Notably, our primary definition of coagulation abnormality (≥1 abnormal parameter out of FIB, D-dimer, and AT-III) pools patients with mild isolated derangements and those with multiple severe abnormalities, leading to within-group heterogeneity. Sensitivity analyses adopting a stricter criterion (≥2 concurrent abnormal markers) and an ordinal composite coagulation score confirmed the robustness of the association between coagulation derangements and adverse IVF outcomes. Live-birth rates declined incrementally as the count of abnormal coagulation markers increased (0: 44.6%, 1: 29.6%, 2: 16.7%, 3: 0.0%; *P* for trend = 0.002), consistent with a dose-response pattern and implying that the true effect size might exceed the estimate derived from our broad primary definition.

The independent associations of elevated D-dimer and reduced AT-III with live birth failure highlight the importance of both enhanced coagulation and compromised anticoagulant capacity. D-dimer, as a marker of fibrin degradation, reflects ongoing activation of both coagulation and fibrinolysis, while AT-III serves as a key endogenous anticoagulant (24, 25). Their combined assessment may provide a more comprehensive evaluation of thrombotic risk than either marker alone. In the hierarchical regression analysis, the associations of D-dimer and AT-III with live birth failure remained statistically significant after adjustment for embryo quality, with the OR for D-dimer attenuated by approximately 16% from Model 2 (OR = 3.412) to Model 3 (OR = 2.864), suggesting that the association between coagulation abnormalities and reproductive outcomes was not fully explained by embryo quality in this dataset. However, because this was an observational study and was not designed to establish causality, this finding should not be interpreted as evidence of a causal biological pathway. Possible contributions of endometrial receptivity and placentation remain speculative.

### 4.1 Study limitations

Several limitations should be acknowledged. First, this was a single-center retrospective study with a relatively modest sample size, which may introduce selection bias and limit generalizability. Second, despite adjustment for key confounders, residual confounding cannot be excluded, particularly for unmeasured factors such as embryonic chromosomal abnormalities, luteal phase insufficiency (26), or uterine structural abnormalities. Third, the primary definition of coagulation abnormalities was based on FIB, D-dimer, and AT-III only. PT, APTT, and TT were analyzed as continuous variables because no validated consensus cutoff exists for defining shortened PT/APTT/TT as a hypercoagulable state. This may underestimate the overall burden of coagulation activation in this population. Although sensitivity analyses using stricter definitions and a composite score yielded consistent findings, statistical power for detailed dose-response analyses was limited. Fourth, because of the observational design, the regression models cannot establish causality. Fifth, patients who developed moderate-to-severe OHSS (*n* = 3) or whose cycles were cancelled (*n* = 3) were excluded after COH initiation. This may have selected patients who successfully progressed to embryo transfer and could have underestimated the association between coagulation abnormalities and live birth failure; the findings are therefore primarily generalizable to women who complete COH and reach embryo transfer. Sixth, EMs could not be completely excluded in the control group because laparoscopy was not routinely performed, and subclinical EMs may have been misclassified as controls; this would tend to attenuate rather than exaggerate between-group differences. Seventh, only the first IVF cycle and first embryo transfer per patient were analyzed. This ensured independence of observations but reduced statistical power and excluded subsequent cycles. Finally, long-term pregnancy outcomes and molecular mechanisms were not assessed. Future multicenter prospective studies with larger sample sizes are needed.

### 4.2 Clinical implications

From a clinical perspective, these findings support further research into coagulation markers in EMs-related infertility. However, this retrospective study cannot establish causality, and D-dimer/AT-III testing should not be adopted into routine pre-IVF clinical decision-making until prospective external validation and randomized trials are available. Current ESHRE guidelines do not recommend routine coagulation testing before IVF in women with endometriosis (27). Recent evidence has also addressed the integration of surgery and IVF in endometriosis-related infertility (28)**.** Any anticoagulation strategy would need to be evaluated against bleeding risks in controlled trial settings (29).

## 5 Conclusion

Infertile patients with endometriosis frequently present with coagulation abnormalities, defined by FIB, D-dimer, and AT-III, that are associated with adverse IVF outcomes. Elevated D-dimer and decreased AT-III were independently associated with live birth failure in this retrospective cohort. These findings are exploratory and require prospective validation. Routine D-dimer and AT-III testing cannot currently be recommended, and randomized trials are needed to determine whether targeted anticoagulation can improve reproductive outcomes.

## Data Availability

The raw data supporting the conclusions of this article will be made available by the authors, without undue reservation.
